# Assessment of the Impacts of Common Morel (*Morchella sextelata*) Cultivation on Soil Physicochemical Properties and Microbial Communities in Different Environments

**DOI:** 10.3390/microorganisms14051115

**Published:** 2026-05-14

**Authors:** Zhongyan Tang, Chen Chen, Li Dong, Liuyuan Bao, Chengcui Yang, Xiaodan Wang, Xiaoling Chen, Xiaokun Li, Fajun Xiang, Shunqiang Yang

**Affiliations:** 1 College of Agronomy and Life Sciences, Zhaotong University, Zhaotong 657000, China; 13988395247@nwsuaf.edu.cn (Z.T.);; 2 Key Laboratory of Innovative Utilization and Cultivation of Edible Fungi Resources, in Yunnan Province, Zhaotong 657000, China; 3 College of Plant Protection, Yunnan Agricultural University, Kunming 650201, China

**Keywords:** *Morchella esculenta*, under apple orchards soil, dryland soil, paddy fields soil, soil chemical properties, microbial diversity, 16S rRNA gene, ITS

## Abstract

*Morchella sextelata* a species of high nutritional and economic value, is widely cultivated. To investigate how different cultivation environments affect the soil physicochemical properties and microbial communities associated with common morel, this study established cultivation plots under three distinct settings: apple orchard canopies, dry upland fields, and paddy fields. The objective was to compare the differential impacts of common morel cultivation on soil environmental conditions across these habitats. The results indicate that cultivating common morel effectively enhances soil fertility. Across all environments, soil hydrolyzable nitrogen (HN), available potassium (AK), and organic matter content were higher than in the control. In apple orchard and dryland soils, total phosphorus (TP), total potassium (TK), available phosphorus (AP), and pH values were also elevated compared to the control, with most differences reaching significant levels. Solid Sucrase (S-SC) activity increased in all environments compared to the control, with values of 17.52 mg/d/g in PG, 17.39 mg/d/g in HD, and 21.68 mg/d/g in DT soils. Soil Amylase (S-AL) activity was higher in PG (451.28 μg/h/g) and HD (475.38 μg/h/g) soils. In contrast, Soil-acid phosphatase (S-ACP) activity was significantly elevated in DT soil (2922.08 nmol/h/g). PG soil exhibited significantly higher activities of Solid-Catalase (S-CAT), Solid polyphenol oxidase (S-PPO), and Solid Urease (S-UE), with S-CAT reaching 952.5 μmol/h/g. Following common morel cultivation, bacterial richness and diversity decreased across all conditions, while fungal richness increased but diversity declined. At the phylum level, *Proteobacteria* remained the dominant bacterial group, accounting for 26.78% in PG, 28.27% in HD, and 20.05% in DT soils. *Ascomycota* was the predominant fungal phylum, comprising 68.03% in PG, 72.16% in HD, and 68.94% in DT soils. Predicted bacterial functional pathways were primarily associated with metabolism, genetic information processing, environmental information processing, and cellular processes. Key metabolic pathways included carbohydrate metabolism, amino acid metabolism, and metabolism of cofactors and vitamins. fungal functional guilds were mainly classified as pathotrophic, pathotrophic–saprotrophic, pathotrophic–saprotrophic–symbiotrophic, and saprotrophic. Among these, saprotrophic and pathotrophic guilds showed higher abundance compared to the control. This shift is characterized by a reduction in both the diversity and abundance of beneficial microorganisms, alongside an increase in the richness of harmful microbial taxa. The combined effect of these factors disrupts the soil microbial equilibrium. The findings of this study provide a theoretical foundation for the cultivation of common morel and the management of associated soils.

## 1. Introduction

*Morchella esculenta*, a rare edible and medicinal fungus belonging to the genus *Morchella* (family Morchellaceae, order Pezizales, phylum Ascomycota) [1,2], is predominantly found in temperate regions [3]. It is highly valued for its distinctive flavor, palatability, and nutritional richness, as well as its antioxidant, antitumor, and immunomodulatory properties [4,5,6,7]. Studies have demonstrated that crop rotation systems—such as *Angelica sinensis*–*Morchella* rotation following seedling cultivation on prepared soil (Ran et al.) and rice–*Morchella* rotation (Duan et al.)—can yield significant economic benefits [8,9].

Soil microorganisms constitute a critical component of terrestrial ecosystems, with their community structure being closely linked to nutrient cycling and plant health [10]. These microorganisms facilitate the transformation of essential elements such as carbon, nitrogen, and phosphorus, thereby enhancing nutrient availability for plants. Conversely, the accumulation of pathogenic microbes in the soil can lead to plant disease and mortality [11]. In contrast, beneficial microorganisms suppress soil-borne pathogens through mechanisms such as antagonism and induced systemic resistance, thereby promoting plant health [12]. Plants can recruit specific microbial taxa to help mitigate environmental stress. In turn, these microbial communities can activate host genes involved in nitrogen uptake under deficient conditions or enhance disease resistance, thereby contributing to stress adaptation [13].

Common morel is typical soil-covered edible fungi that rely on external nutrient bags for nourishment, completing their entire growth cycle within the soil [14]. Previous studies have demonstrated that Common morel species exhibit a substantial uptake of available phosphorus during growth, while simultaneously enriching total potassium in soil, thereby enhancing overall soil fertility [15]. Endophytic bacteria within common morel, particularly from the phyla Bacteroidota, Pseudomonadota, and Bacillota, have been shown to significantly influence its growth [16]. The cultivation of common morel leads to a significant increase in both the diversity index and abundance of soil bacteria compared to control groups [17]. The 10–20 cm soil layer demonstrates higher microbial diversity and is notably rich in nitrogen-fixing bacteria [18]. Soil physicochemical properties and microbial composition play crucial roles in mycelial growth, primordium differentiation, and fruiting body formation [19]. In the cultivation and management of common morel, practices such as adjusting soil pH, increasing soil organic matter content, and applying microbial agents—including *Bacillus subtilis* and *Lysinibacillus fusiformis*—are employed to promote mycelial growth and prevent disease. Soil microorganisms play a key role in this context [20]. Despite these advances in elucidating the impacts of the soil microbiome, a comprehensive understanding of its dynamics following common morel cultivation across different environments—namely under apple orchards, in dryland, and in paddy fields—remains lacking. In this study, we employed 16S rRNA and ITS amplicon sequencing to characterize the functional composition of the soil microbial community. Specifically, we aimed to investigate (1) the effects of common morel cultivation on soil physicochemical properties across different environments; (2) the richness, diversity, and composition of microbial communities following common morel cultivation in these environments; and (3) the functional attributes of soil microbes and their inferred associations with soil fertility and health.

## 2. Materials and Methods

### 2.1. Materials

**Figure 1:**
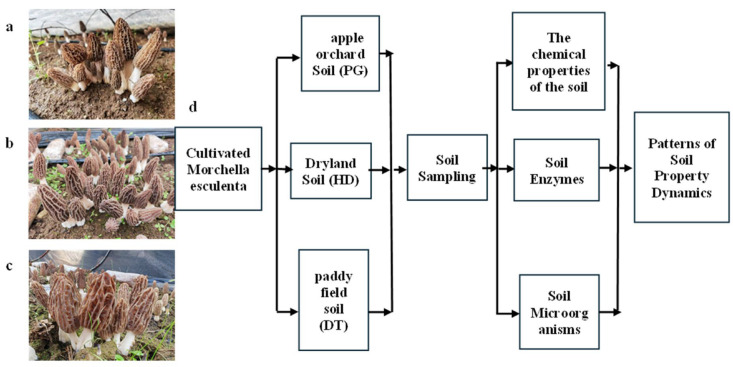
Experimental design for the cultivation of common morel in paddy fields and dry lands during the fallow period. (**a**) Common morel cultivated in paddy soil (DT soils). (**b**) Common morel cultivated in dryland soils (HD soils). (**c**) Common morel cultivated in paddy field soil (DT soils). (**d**) Technical outline of this study.

Common morel was cultivated in three distinct environments: between apple tree rows in Yina Village (Figure 1a; 27.29° N, 103.55° E, at an elevation of 1899.20 m), on dryland (previously planted with maize) in Shuanghe Village (Figure 1b), and in a paddy field (27.27° N, 103.60° E, at an elevation of 1400.25 m) in Zhaoyang District, Yunnan Province, China (Figure 1c). Common morel (Variety name: *Morchella sextelata*, obtained from Longxing Biotechnology Co., Ltd., Zhaotong, China) was cultivated in November 2023 and harvested in March–April 2024.

### 2.2. Methods

#### 2.2.1. Soil Sample Collection

From March to April 2024, for each cultivation environment, post-cultivation soil was collected alongside control soil from the same environment where the common morel was not grown. The 0–20 cm tillage layer soil was sampled using the five-point sampling method. After removing debris, one portion of each sample was stored at −86 °C in sterile cryotubes for microbial analysis. The remaining soil was air-dried, ground, sieved, stored in sealed bags protected from light, and subsequently used for analysis of soil chemical properties and enzyme activities [11].

#### 2.2.2. Analysis of Soil Chemical Properties

Total soluble sugar content in soil was determined using the anthrone colorimetric method; ssoil pH was measured using the potentiometric method as described by Chen Yu, with a soil-to-water ratio of 2.5:1 [21]; electrical conductivity was determined using a conductivity meter at a soil-to-water ratio of 5:1 [22]; total nitrogen (TN) content was determined using the Kjeldahl method following digestion with sulfuric acid and an accelerator [23]; total phosphorus (TP) content was determined using the molybdenum-antimony anti-spectrophotometric method after NaOH alkali fusion [24]; total potassium (TK) content was measured using a flame photometer following NaOH alkali fusion [25]; alkali-hydrolyzable nitrogen (HN) content was determined using the alkali diffusion method after NaOH hydrolysis; available phosphorus (AP) content was determined using the molybdenum-antimony colorimetric method after extraction with ammonium fluoride-hydrochloric acid and sodium bicarbonate solutions [22]; and available potassium (AK) content was measured using a flame photometer following ammonium acetate extraction [26].

#### 2.2.3. Determination of Soil Enzyme Activities

Soil enzyme activities were measured using a microplate reader assay. Commercial assay kits (Griess Biotechnology Co., Ltd., Suzhou, China) were used to determine the activities of Soil Amylase (S-AL), Soild- Polyphenol oxidase (S-PPO), Soil catalase (S-CAT), Soil Urease (S-UE), Soil acid phosphatase (S-ACP), and Soil Sucrase (S-SC) in air-dried soil samples. All soil samples were air-dried, passed through a 25-mesh sieve, and analyzed with three replicates per treatment.S-AL: Soil Amylase, primarily derived from microorganisms, is a key enzymatic preparation. It hydrolyzes starch to produce reducing sugars, which react with 3,5-dinitrosalicylic acid to form a brownish-red compound with a characteristic absorption peak at 540 nm, allowing for the determination of S-AL. One unit of S-AL is defined as the amount of enzyme that catalyzes the production of 1 μg of glucose per gram of soil per hour. S-PPO: S-PPO catalyzes the oxidation of the substrate L-DOPA to produce red quinone compounds. These colored products exhibit a characteristic absorbance peak at 475 nm, which is measured to calculate the enzyme activity. One unit of S-PPO activity is defined as the amount of enzyme that produces 1 nmol of the red product per gram of soil per hour. S-UE: S-UE activity was measured using the indophenol blue colorimetric method. S-UE hydrolyzes urea to produce ammonia nitrogen (NH_3_-N), which reacts with hypochlorite and phenol under strongly alkaline conditions to form the water-soluble dye indophenol blue. This compound exhibits maximum absorbance at 578 nm, with the color intensity being directly proportional to the NH_3_-N concentration in the solution, thus reflecting S-UE activity. One unit of S-UE activity is defined as the amount of enzyme that produces 1 μg of NH_3_-N per gram of soil per day. S-ACP Activity: Under acidic conditions, S-ACP catalyzes the hydrolysis of p-nitrophenyl phosphate (PNPP) to produce yellow p-nitrophenol (PNP), which has a maximum absorbance peak at 405 nm. The S-ACP activity is determined by measuring the rate of increase in PNP absorbance at this wavelength. One unit of S-ACP activity is defined as the amount of enzyme that hydrolyzes PNPP to produce 1 nmol of PNP per gram of soil per hour. S-CAT Activity: S-CAT primarily decomposes hydrogen peroxide in the soil, thereby mitigating the damage caused by its excessive accumulation to plant roots. CAT catalyzes the breakdown of hydrogen peroxide into water and oxygen. The residual hydrogen peroxide reacts with a highly sensitive chromogenic probe to produce a colored compound with a maximum absorbance peak near 510 nm. The CAT activity is calculated based on the decrease in hydrogen peroxide concentration. One unit of S-CAT activity is defined as the amount of enzyme that catalyzes the degradation of 1 μmol of H_2_O_2_ per gram of soil per hour. S-SC Activity: S-SC activity was determined using the DNS colorimetric method. S-SC catalyzes the degradation of sucrose to produce reducing sugars, which subsequently react with 3,5-dinitrosalicylic acid to form colored amino compounds with a characteristic absorbance peak at 540 nm. Within a certain range, the rate of increase in absorbance at 540 nm is directly proportional to the S-SC activity, allowing for its quantification. One unit of S-SC activity is defined as the amount of enzyme that produces 1 mg of glucose per gram of soil per day.

#### 2.2.4. Analysis of Soil Microbiota

DNA was extracted from soil samples using the HiPure Soil DNA Kit (Magen, Guangzhou, China) following the manufacturer’s protocol [27]. To comprehensively assess the diversity of both bacterial and fungal communities, universal primers were employed. The primers 341F (5′-CCTACGGGNGGCWGCAG-3′) and 806R (5′-GGACTACHVGGGTATCTAAT-3′) were used to amplify the 16S rDNA V3-V4 region in the ribosomal RNA gene and the primers ITS3_KYO2: 5′-GATGAAGAACGYAGYRAA-3′ and ITS4: 5′-TCCTCCGCTTATTGATATGC-3′ were used to amplify the Internal Transcribed Spacer ITS2 region in the ribosomal RNA gene for polymerase chain reaction (PCR) [28]. The target region of the bacterial 16S rRNA gene and ITS were amplified under the following conditions: initial denaturation at 95 °C for 5 min; 30 cycles of denaturation at 95 °C for 1 min, annealing at 60 °C for 1 min, and extension at 72 °C for 1 min; followed by a final extension at 72 °C for 7 min. The 50 μL reaction mixture consisted of 10 μL 5× Q5^®^ Reaction Buffer, 10 μL 5× Q5^®^ High GC Enhancer, 1.5 μL of 2.5 mM dNTPs, 1.5 μL each of forward and reverse primers (10 μM), 0.2 μL Q5^®^ High-Fidelity DNA Polymerase, and 50 ng of template DNA [29]. PCR reagents were obtained from New England Biolabs (Ipswich, MA, USA). The quality of PCR amplicons was assessed on a 2% agarose gel. Products were purified using AMPure XP Beads (Beckman, CA, USA) and quantified with a Qubit 3.0 Fluorometer. Sequencing libraries were prepared using the Illumina DNA Prep Kit (Illumina, CA, USA). Library quality was checked using an ABI StepOnePlus Real-Time PCR System (Life Technologies, Foster City, CA, USA). Qualified libraries were pooled and sequenced on an Illumina NovaSeq 6000 platform using a PE250 strategy [30].

#### 2.2.5. Data Analysis

Statistical analyses were performed using Excel 2019 and IBM SPSS Statistics 27. Graphical representations of the results were generated with Origin 2024. For the sequencing data, raw reads from the Illumina platform were filtered using FASTP (version 0.18.0) to obtain clean reads for subsequent analysis. Clean reads were merged into tags using FLASH (version 1.2.11), with a minimum overlap of 10 bp and a maximum mismatch rate of 2%. The clean tags were subsequently clustered into operational taxonomic units (OTUs) using the UPARSE algorithm in Usearch (version 11.0.667), based on a 97% similarity threshold. Taxon abundance statistics were visualized using Krona (version 2.6). Stacked bar plots illustrating species abundance were generated with the ggplot2 package in R, while heatmaps of species abundance were produced using the pheatmap package in R software (R 4.5.0). For indicator species analysis, Venn diagrams depicting shared and unique species among groups were constructed using the VennDiagram package in R software. Differences in alpha diversity indices between two groups and among multiple groups were assessed using the vegan package in R software. For 16S rRNA gene amplicon data, functional profiles of Kyoto Encyclopedia of Genes and Genomes (KEGG) metabolic pathways were predicted using PICRUSt2 (version 2.5.3). For ITS data, the functional guilds of fungi were inferred using FUNGuild (version 1.1).

## 3. Results and Analysis

### 3.1. Effects of Cultivating Common Morel in Different Environments on Soil Chemical Properties

Cultivating common morel under different conditions variably affected soil chemical properties, depending on the specific soil environment (Table 1). In the apple orchard, following common morel cultivation, the levels of total phosphorus (TP), total potassium (TK), hydrolyzable nitrogen (HN), available phosphorus (AP), available potassium (AK), organic matter, pH, and EC were all higher than those in the control. These increases were statistically significant, with the exception of HN. In contrast, total nitrogen (TN) was significantly lower than in the control. In the dryland field, the contents of TP, TK, HN, AP, AK, organic matter, and pH were significantly higher than those in the control, while TN and EC were lower. In the paddy field, the contents of HN, AK, and organic matter were higher than those in the control, whereas TN, TP, TK, AP, pH, and EC were lower. Notably, across all three environments, soil TN content was consistently lower than that in the respective control groups. Following cultivation, soil TN content decreased relative to the control in all settings, with significant reductions observed in PG soil (1.40 g/kg) and DT soil (1.64 g/kg). In contrast, levels of HN, AK, and Organic Matter increased compared to the control. The increases in HN were significant in the HD soil (153.62 mg/kg) and DT soil (193.31 mg/kg). Significant increases in AK were found in the PG soil (482.54 mg/kg) and HD soil (209.24 mg/kg). Similarly, organic matter content increased significantly in the PG soil (35.15 g/kg) and DT soil (38.85 g/kg). For soils in the PG and HD soils, TP, TK, AP, and pH values were generally higher post-cultivation than in the control, with most differences being significant. EC was significantly lower than the control in the HD (136.13 μS/cm) and DT (170.79 μS/cm).

### 3.2. Effects of Common Morel Cultivation in Different Environments on Soil Enzyme Activities

The impact of common morel cultivation on soil enzyme activities varied significantly depending on the soil environment (Table 2). Following cultivation, the activities of S-CAT, S-PPO, and S-UE were significantly higher in PG soil than in the control, with S-CAT activity reaching 952.5 μmol/h/g; in contrast, activities in HD and DT soils were lower than the control. Both PG and HD soils showed higher activities of S-AL and S-SC compared to the control, with most differences being significant. The S-AL activity in these environments was 475.38 μg/h/g. In DT soil, S-SC activity (21.68 mg/d/g) was higher than the control, while S-AL activity (200.88 μg/h/g) was lower. S-ACP activity was significantly elevated in PG soil (2922.08 nmol/h/g) but was significantly reduced in both PG (1487.90 nmol/h/g) and HD (1164.14 nmol/h/g) soils relative to the control.

### 3.3. Comparison of Rhizosphere Microorganisms in Soils from Different Environments

#### 3.3.1. Operational Taxonomic Units (OTUs) Analysis of Rhizosphere Microorganisms in Soils from Different Environments

**Figure 2:**
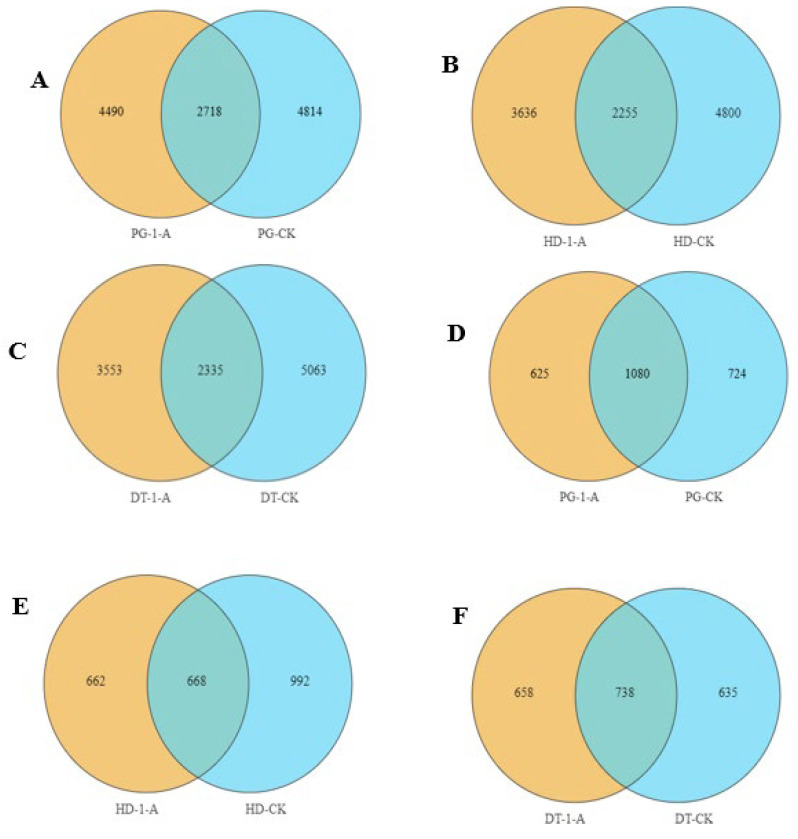
Venn diagrams illustrating the bacterial and fungal operational taxonomic units (OTUs) in soils from different cultivation environments. (**A**) Venn diagram illustrating the operational taxonomic units (OTUs) of soil bacteria following the cultivation of common morel in PG soils; (**B**) Venn diagram illustrating the operational taxonomic units (OTUs) of soil bacteria following the cultivation of common morel in HD soils; (**C**) Venn diagram illustrating the operational taxonomic units (OTUs) of soil bacteria following the cultivation of common morel in DT soils; (**D**) Venn diagram illustrating fungal OTUs distributions in soil following the cultivation of Common morel in PG soils; (**E**) Venn diagram illustrating fungal OTUs distributions in soil following the cultivation of common morel in HD soils; and (**F**) Venn diagram illustrating fungal OTUs distributions in soil following the cultivation of common morel in DT soils.

The numbers of unique bacterial sequences in soils planted with common morel was 4490, 3636, and 3553 for the PG (Figure 2A), HD (Figure 2B), and DT (Figure 2C) environments, respectively, while the unique fungal sequences were 625 (Figure 2D), 662 (Figure 2E), and 658 (Figure 2F), respectively. In contrast, unplanted soils from the same environments contained 4814 (Figure 2A), 4800 (Figure 2B), and 5063 (Figure 2C) unique bacterial sequences and 724 (Figure 2D), 992 (Figure 2E), and 635 (Figure 2F) unique fungal sequences, respectively. The results indicate that planting common morel reduced the number of unique bacterial sequences across all environments. For fungi, unique sequences decreased in the PG and HD but increased in the DT soils.

#### 3.3.2. Analysis of Relative Abundance of Rhizosphere Microbiota in Different Soils

**Figure 3:**
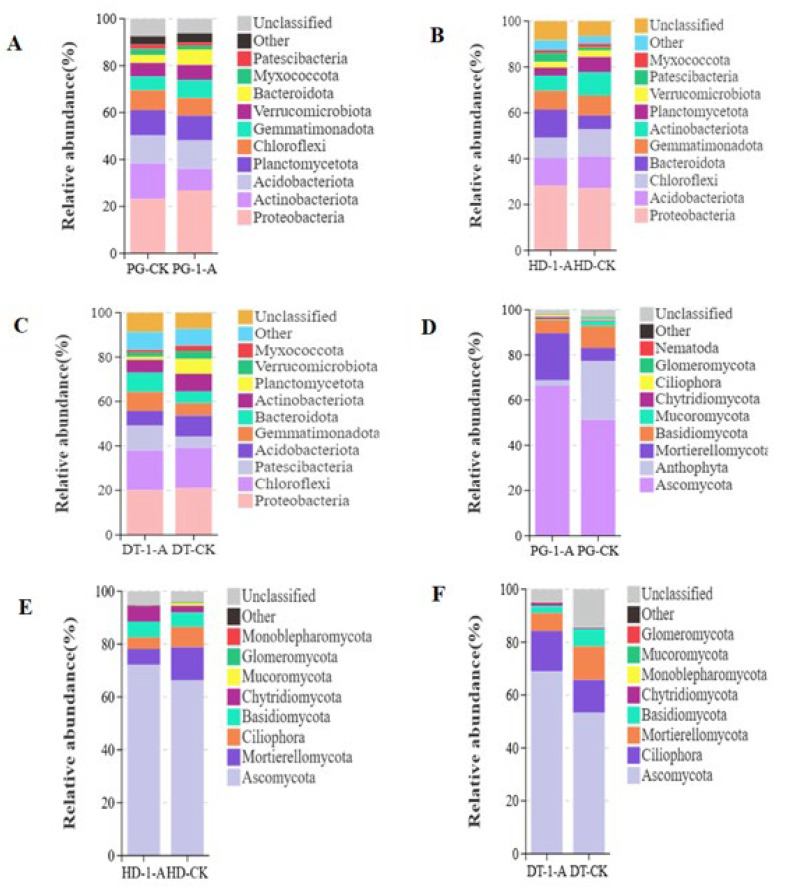
The relative abundance of dominant bacterial and fungal communities at the phylum level. (**A**) Bacterial community composition at the phylum level in soils under common morel cultivation in PG soils; (**B**) bacterial community composition at the phylum level in soils under common morel cultivation in HD soils; (**C**) bacterial community composition at the phylum level in soils under common morel cultivation in DT soils; (**D**) fungal community composition at the phylum level in soil under common morel cultivation in PG soils; (**E**) fungal community composition at the phylum level in soil under common morel cultivation in HD soils; (**F**) fungal community composition at the phylum level in soil under common morel cultivation in DT soils.

Proteobacteria was the dominant bacterial phylum in soils after common morel cultivation across all environments, with relative abundances of 26.78% (PG-1-A), 28.27% (HD-1-A), and 20.05% (DT-1-A). The relative abundances of specific phyla were higher in cultivated soils compared to their respective controls: Proteobacteria, Acidobacteriota, and Gemmatimonadota in the PG soil (Figure 3A); Proteobacteria, Bacteroidota and Patescibacteria in the HD soil (Figure 3B); Patescibacteria, Gemmatimonadota, and Bacteroidota in the DT soil (Figure 3C). The abundances of other phyla showed the opposite trend. Notably, the Bacteroidota increased across all environments, while Proteobacteria increased specifically in the PG and HD soils, and Gemmatimonadota increased in the PG and DT soils. Conversely, several phyla exhibited decreased abundance in cultivated soils, including Actinobacteriota, Planctomycetota, Chloroflexi, and Myxococcota across all environments, as well as Acidobacteriota and Verrucomicrobiota in the HD and DT soils.

Ascomycota was the dominant fungal phylum in soils from all environments following common morel cultivation, with relative abundances of 68.03% (PG-1-A), 72.16% (HD-1-A), and 68.94% (DT-1-A), respectively. The relative abundances of Ascomycota, Mortierellomycota, Chytridiomycota, and Ciliophora in the PG soil (Figure 3D); Ascomycota, Chytridiomycota, and Basidiomycota in the HD soil (Figure 3E); and Ascomycota, Ciliophora, Chytridiomycota, and Mucoromycota in the DT soil were higher than those in the control, while the relative abundances of other phyla were lower (Figure 3F). Specifically, the relative abundances of Chytridiomycota and Ascomycota across all environments, as well as Ciliophora in PG and DT soils, were higher than in the control. In contrast, the relative abundances of Glomeromycota across all environments, Basidiomycota in PG and DT soils, Mucoromycota in PG and HD soils, and Mortierellomycota in HD and DT soils were lower than the control.

**Figure 4:**
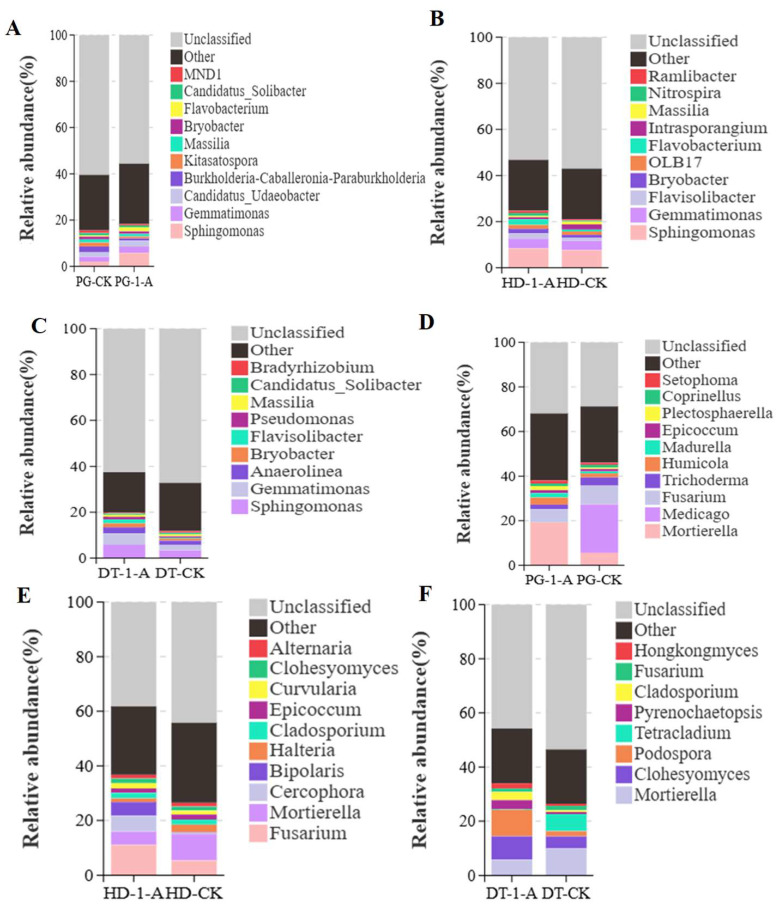
Relative abundance of dominant bacterial and fungal genera. (**A**) Bacterial community composition at the genus level in soil under common morel cultivation in PG soils; (**B**) bacterial community composition at the genus level in soil under common morel cultivation in HD soils; (**C**) bacterial community composition at the genus level in soil under common morel cultivation in DT soils; (**D**) fungal community composition at the genus level in soil under common morel cultivation in PG soils; (**E**) fungal community composition at the genus level in soil under common morel cultivation in PG soils; (**F**) fungal community composition at the genus level in soil under common morel cultivation in PG soils.

*Sphingomonas* was the dominant genus in soils from all environments following common morel cultivation, with relative abundances of 5.75% (PG-1-A), 8.38% (HD-1-A), and 5.79% (DT-1-A). Compared to the control, several genera showed increased relative abundance. In PG soil, these were *Sphingomonas*, *Gemmatimonas*, *Candidatus_Udaeobacter*, *Flavisolibacter*, *Candidatus_Solibacter*, and *MND1* (Figure 4A). In HD soil, the enriched genera included *Sphingomonas*, *Gemmatimonas*, *Flavisolibacter*, *Bryobacter*, *OLB17*, *Flavobacterium*, *Nitrospira*, and *Ramlibacter* (Figure 4B). In DT soil, *Sphingomonas*, *Gemmatimonas*, *Anaerolinea*, *Bryobacter*, *Flavisolibacter*, *Pseudomonas*, and *Massilia* were more abundant (Figure 4C). All other genera exhibited lower abundance than the control. Specifically, *Sphingomonas* and *Gemmatimonas* were enriched across all three cultivation environments. *Flavobacterium* was enriched in both apple orchard and dryland soils, *Flavisolibacter* was enriched in apple orchard and paddy field soils, and *Bryobacter* was enriched in HD and DT soils. Conversely, *Massilia* showed lower relative abundance in PG and HD soils, and *Candidatus_Solibacter* was less abundant in PG and DT soils compared to the control.

The predominant fungal genera identified in soils following common morel cultivation were *Mortierella* (19.22%) in the PG soil, *Fusarium* (10.99%) in the HD soil, and *Podospora* (9.55%) in the DT soil. Relative to the control, an increased relative abundance was observed for the following genera: *Mortierella*, *Humicola*, *Madurella*, *Epicoccum*, *Plectosphaerella*, and *Setophoma* in the PG soil (Figure 4D); *Fusarium*, *Cercophora*, *Bipolaris*, *Cladosporium*, *Curvularia*, and *Clohesyomyces* in the HD soil (Figure 4E); *Clohesyomyces*, *Podospora*, *Pyrenochaetopsis*, *Cladosporium*, and *Hongkongmyces* in the DT soil (Figure 4F). All other genera exhibited lower abundances compared to the control. Notably, the relative abundances of *Clohesyomyces* and *Cladosporium* were elevated in both the HD and DT soils.

#### 3.3.3. Analysis of Rhizosphere Soil Microbial Alpha Diversity Under Different Environmental Conditions for Common Morel Cultivation

Alpha diversity indices were used to assess the richness and diversity of bacterial and fungal communities following common morel cultivation in three environments: PG, HD, and DT soils. Table 3 the shows that the sequencing coverage was high, with bacterial community coverage reaching 99.6% to 99.7% and fungal community coverage at 100% across all conditions, indicating reliable and comprehensive data. Following common morel cultivation, bacterial richness decreased across all environments compared to their respective controls. Bacterial diversity decreased in the PG and DT soils but increased in the HD soil relative to controls. In contrast, fungal richness increased in the PG and DT soils but decreased in the HD soil. Fungal diversity decreased in the PG and HD soils, while it increased in the DT soil. Collectively, these results indicate that common morel cultivation generally reduces bacterial community richness, increases fungal community richness, and tends to decrease the diversity of both bacterial and fungal communities.

#### 3.3.4. Analysis of Soil Rhizospheric Microbial Beta Diversity Across Different Environments

**Figure 5:**
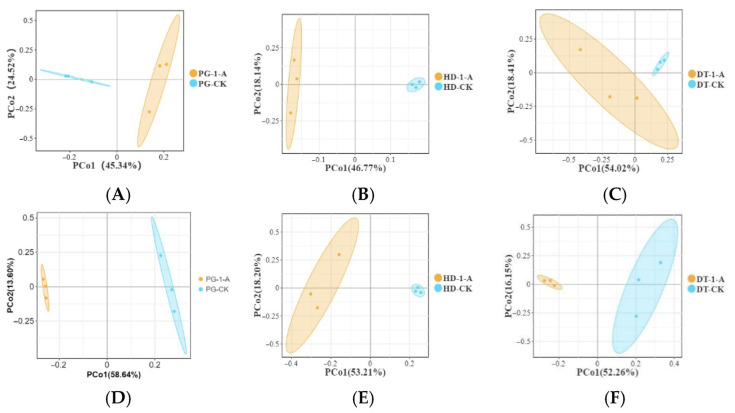
(**A**) The PCoA scatter plot illustrates the bacterial community composition in soil where ME was cultivated in PG soils; (**B**) the PCoA scatter plot illustrates the bacterial community composition in soil where ME was cultivated in HD soils; (**C**) the PCoA scatter plot illustrates the bacterial community composition in soil where ME was cultivated in DT soils; (**D**) the PCoA scatter plot illustrates the fungal community composition in soil where ME was cultivated inn PG soils; (**E**) the PCoA scatter plot illustrates the fungal community composition in soil where ME was cultivated in PG HD soils; (**F**) the PCoA scatter plot illustrates the fungal community composition in soil where ME was cultivated in DT soils.

To assess the impact of ME cultivation on bacterial community composition in different environments, beta diversity was analyzed. Principal Coordinate Analysis (PCoA) based on OTU-level data was performed on soils both before and after ME introduction. The variances explained by the first two principal coordinates were as follows: for the PG soil (Figure 5A), the first axis (PCo1) accounted for 45.34% and the second axis (PCo2) for 24.52%; for HD soil (Figure 5B), PCo1 explained 46.77% and PCo2 explained 18.14%; and for DT soil (Figure 5C), PCo1 explained 54.02% and PCo2 explained 18.41%. Separation along the PCo1 was observed between pre- and post-cultivation bacterial communities across all environments. PERMANOVA (Adonis) tests indicated that while bacterial community structure in PG and HD soils showed changes after ME cultivation, these differences were not statistically significant (AG soil: R^2^ = 0.4361, *p* = 0.1; HD soil: R^2^ = 0.3064, *p* = 0.1). PCo1 and PCo2 of the fungal community in the PG soil explained 58.64% and 13.60% of the variation, respectively (Figure 5D). For the fungal community in HD soil (Figure 5E), PCo1 and PCo2 accounted for 53.21% and 18.20% of the variation, respectively. In the overall fungal community, PCo1 and PCo2 explained 52.26% and 16.15% of the variation, respectively. Along PCo1, a separation was observed between the fungal community compositions of soils before and after ME cultivation across different environmental conditions. Adonis (PERMANOVA) tests indicated that the fungal community structures in the three soil environments changed after ME cultivation, although the differences were not statistically significant (RPG2 = 0.4361, PPG = 0.1; RHD2 = 0.3064, PHD = 0.1).

#### 3.3.5. Predictive Functional Analysis of Rhizosphere Soil Microbiota Across Environments

**Figure 6:**
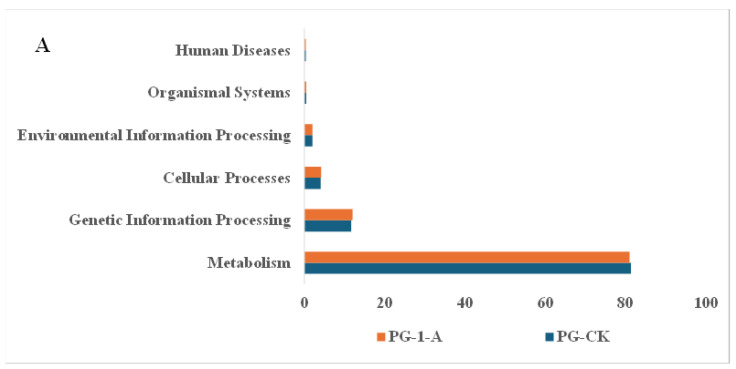

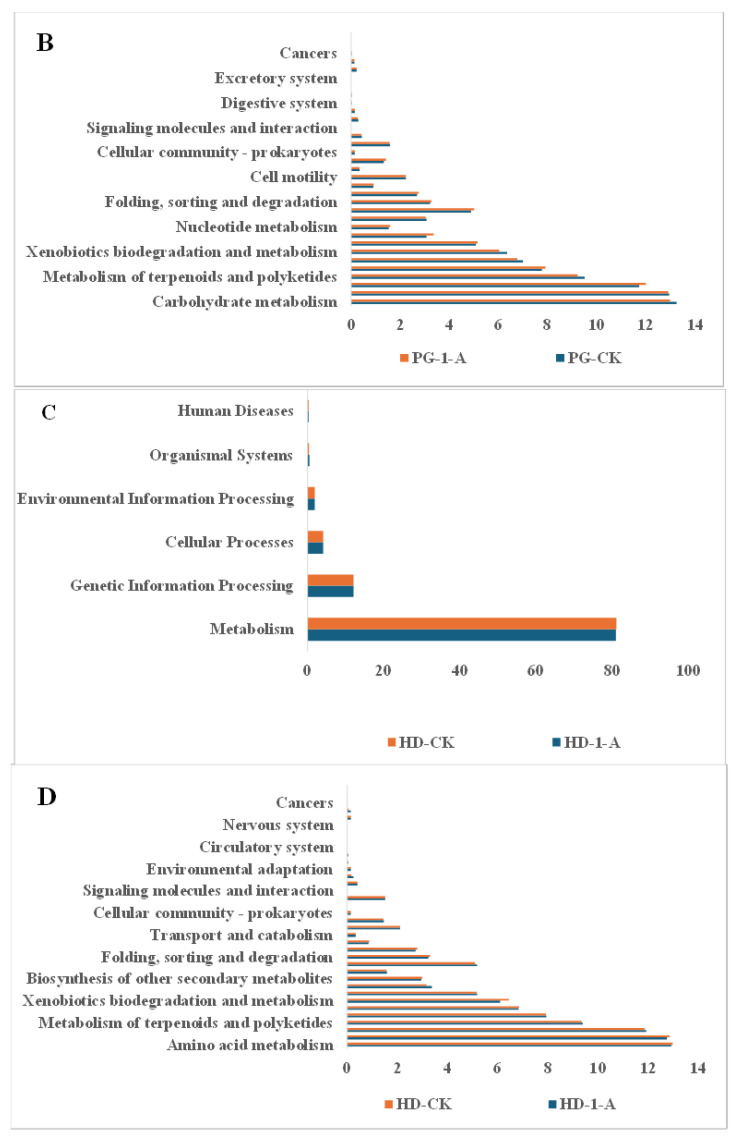

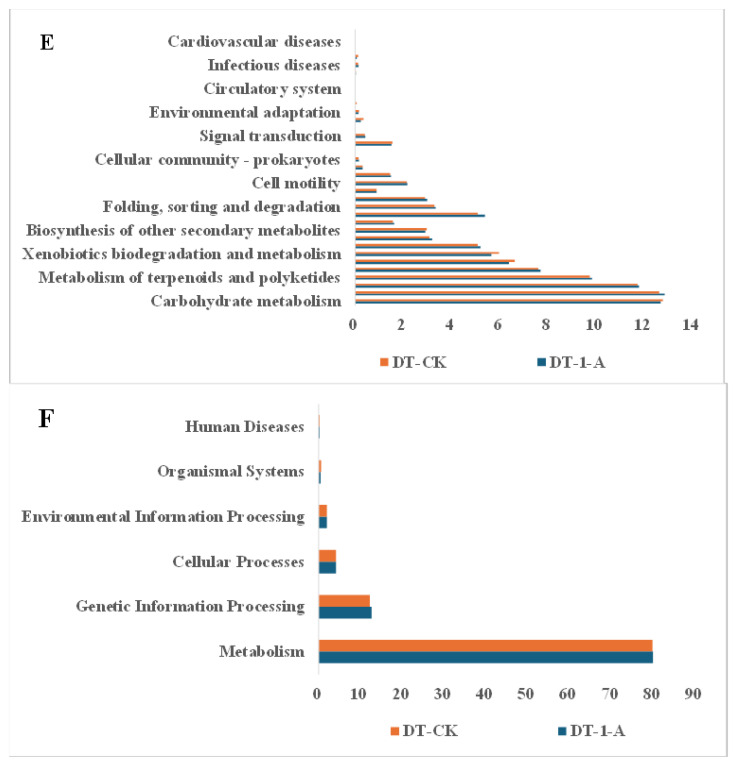
Predicting the functional profiles of soil bacterial communities after morel mushroom cultivation across diverse agricultural systems. (**A**) Functional prediction of soil bacterial communities at the primary hierarchy in common morel cultivation in PG soils; (**B**) functional prediction of soil bacterial communities at the secondary hierarchy level in common morel cultivation in PG soils; (**C**) functional prediction of soil bacterial communities at the primary hierarchy in common morel cultivation in HD soils; (**D**) functional prediction of soil bacterial communities at the secondary hierarchy level in common morel cultivation in HD soils; (**E**) functional prediction of soil bacterial communities at the primary hierarchy in common morel cultivation in DT soils; (**F**) Functional prediction of soil bacterial communities at the secondary hierarchy level in common morel cultivation in DT soils.

The functional potential of the soil bacterial communities across different environments was predicted using PICRUSt2 (Figure 6A–F). At Level 1, six primary metabolic pathways were identified. Common morel metabolism was the most abundant pathway, comprising 80.98–81.34% (PG), 81.09–81.25% (HD), and 79.77–79.80% (DT) of the predicted functions. This was followed by genetic information processing (11.98–11.69% PG, 4.07–4.16% HD, 2.00–2.02% DT), cellular processes (12.05–12.11% PG, 4.10–4.07% HD, 1.94–1.96% DT), and environmental information processing (11.69–11.98% PG, 4.07–4.16% HD, 2.02–2.00% DT). Pathways for human diseases and organismal systems each accounted for less than 1%. Within the Level 2 sub-functions under the dominant metabolism category, the most enriched functional abundances following common morel cultivation across environments were related to carbohydrate metabolism, amino acid metabolism, and metabolism of cofactors and vitamins.

**Figure 7:**
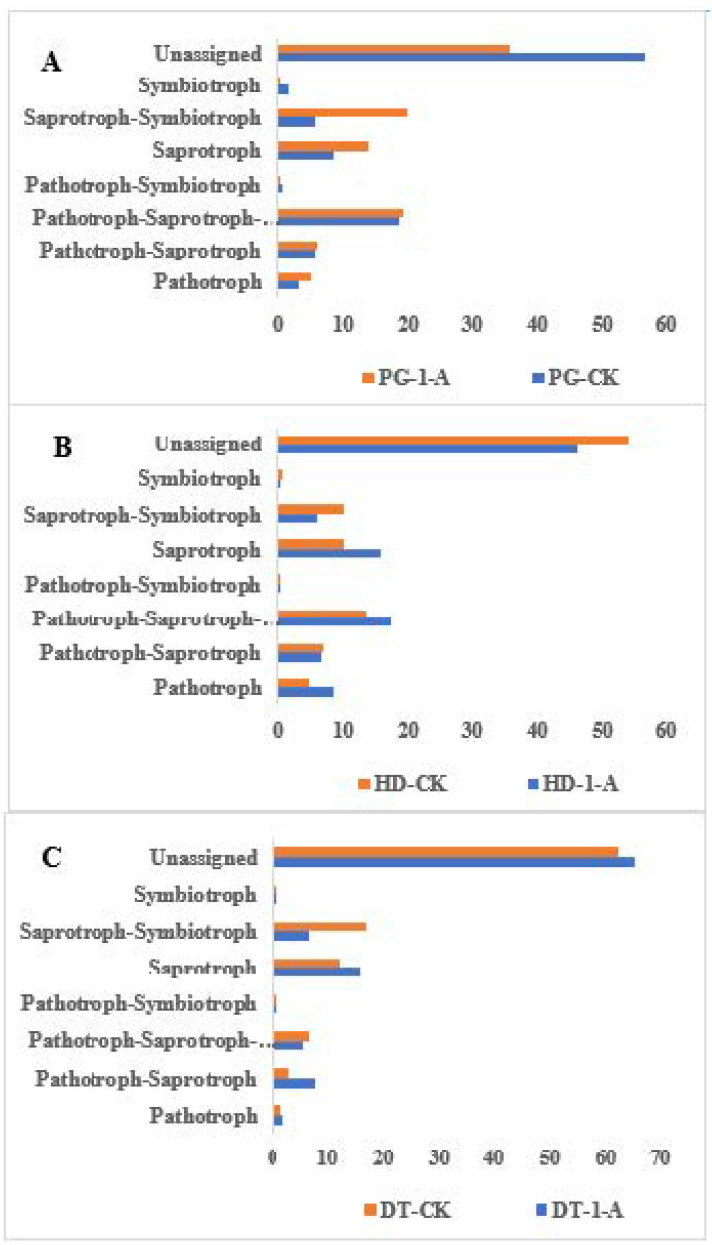
Predicted functions of soil fungal communities after *Morchella* cultivation across various environments. (**A**) Functional Prediction of soil fungal communities in common morel cultivation in PG soils; (**B**) functional prediction of soil fungal communities in common morel cultivation in HD soils; (**C**) functional prediction of soil fungal communities in common morel cultivation in DT soils.

Fungal functional guilds in soils from different environments were analyzed using FunGuild (Figure 7A–C). The soil fungi were classified into three primary trophic modes: saprotroph, pathotroph, and symbiotroph. Additionally, several overlapping trophic modes were identified: pathotroph–saprotroph, pathotroph–saprotroph–symbiotroph, pathotroph–symbiotroph, and saprotroph–symbiotroph. Across all environments, the saprotroph–symbiotroph guild was the most abundant. The predominant guilds varied by environment, including pathotroph, pathotroph–saprotroph, pathotroph–saprotroph-symbiotroph, and saprotroph. Further comparative analysis revealed that the relative abundances of pathotroph, pathotroph–saprotroph, and saprotroph guilds were higher across the experimental environments than in the control. In soils from PG and HD, the pathotroph-saprotroph-symbiotroph and saprotroph-symbiotroph guilds also exhibited higher abundances compared to the control. In contrast, the pathotroph–symbiotroph and symbiotroph guilds showed lower abundances relative to the control. Specifically in DT soil, the relative abundances of the pathotroph–saprotroph–symbiotroph, pathotroph–symbiotroph, and saprotroph–symbiotroph guilds were all lower than those in the control.

## 4. Discussion

### 4.1. Effects of Planting Morchella spp. in Different Environments on Soil Physicochemical Properties

The growth and development of Common morel are favored in well-drained, organically rich soils with a pH of 6.5–7.5. Abundant nitrogen sources facilitate rapid mycelial growth and proliferation, adequate phosphorus promotes thallus development, and potassium enhances mycelial stress resistance [23,31]. Consequently, its growth is differentially impacted by environmental conditions. Previous studies have reported increases in soil pH, organic matter, total nitrogen, alkali-hydrolyzable nitrogen, available phosphorus, and total potassium following common morel cultivation [30,32,33]. Peach–common-morel intercropping significantly elevates soil organic matter, available potassium, and available zinc [29]. Additionally, soil electrical conductivity (EC) decreases notably after cultivation in arid land and paddy fields [20]. In the present study, soil hydrolyzable nitrogen (HN), available potassium (AK), and organic matter content were higher in common-morel-cultivated soils (paddy fields, apple orchards, arid land) compared to controls. One possible explanation is that, during common morel cultivation, the mycelium accelerates the decomposition of lignin and cellulose, along with the degradation of its own mycelial biomass, thereby increasing soil organic matter content. Additionally, common morel cultivation alters soil enzyme activity, which promotes the release of soil mineral elements such as phosphorus and potassium. Consequently, the levels of total phosphorus (TP), total potassium (TK), and available phosphorus (AP) in the soil increase, enhancing soil fertility. These findings are consistent with those of previous studies [34]. Furthermore, the mycelia secrete alkaline amines, such as N,N′-(pentane-1,5-diyl)diacetamide, during growth, raising soil pH [19]. This elevated pH increases phosphorus availability and promotes nitrification, thereby boosting levels of available phosphorus and nitrate nitrogen. Given the more substantial increase in soil nutrients observed in apple orchards and arid land systems in this study, it is plausible that common morel cultivation exerts a more pronounced soil-amending effect in these habitats.

### 4.2. Effects of Morchella Cultivation in Different Environments on Soil Enzyme Activity

Soil enzymes act as catalysts for biochemical reactions and participate in the biogeochemical cycling of various elements, serving as effective indicators of soil fertility [35]. This study found that sucrase activity was elevated across all environments compared to the control. In soils from apple orchards and arid land, amylase activity was higher. Notably, acidic phosphatase activity was significantly higher in paddy field soil, while catalase, polyphenol oxidase, and urease activities were significantly higher in apple orchard soil. Previous research has shown that Common morel cultivation significantly enhances phosphatase activity in the plant rhizosphere soil [36], a finding consistent with the enzyme activity changes observed in apple orchard and arid land soils in this study. A study by Zhao et al. reported a trend of soil amylase activity decreasing, then increasing, and subsequently decreasing again [37]. The discrepancy with our results may be attributed to starch being a primary carbon source for common morel [38]. During peak growth phases with high mycelial vigor and nutrient demand, the fungus likely secretes more hydrolytic enzymes. Consequently, the activities of sucrase, amylase, acidic phosphatase, catalase, polyphenol oxidase, and urease increase synergistically to support robust fungal growth. Mechanistically, amylase facilitates the carbon cycle by catalyzing the hydrolysis of complex starch into smaller carbohydrates [39]. Sucrase catalyzes the hydrolysis of sucrose to glucose and fructose, while amylase breaks down starch into monosaccharides, thereby enhancing the transformation of soil organic matter [40]. Acidic phosphatase increases soil phosphorus availability and is significantly correlated with soil carbon, nitrogen, available phosphorus content, and pH [20]. Catalase catalyzes redox reactions and the degradation of harmful substances in soil. Elevated catalase activity can enhance the intensity of humification and the rate of organic matter transformation and is linked to the abundance and activity of soil microorganisms [10,41,42]. Increased urease activity accelerates the conversion rates of soil total nitrogen, ammonium nitrogen, and nitrate nitrogen [43].

### 4.3. Effects of Cultivating Morchella in Different Environments on Soil Microbial Communities

Soil microorganisms are the primary drivers of soil metabolism. The richness and diversity of soil microbial communities influence the composition and content of soil metabolites, and conversely, soil metabolites can affect the structural composition and abundance of microbial communities by altering the rhizosphere microenvironment [44]. In this study, common morel cultivation across different environments reduced the richness of bacterial communities while increasing that of fungal communities, and it decreased the diversity of both bacterial and fungal communities. Given that certain soil fungi can act as plant pathogens, it is inferred that cultivating morels under apple trees and in dryland conditions may promote soil health by reducing potentially harmful fungal diversity. This finding aligns with Chuan Xiong et al. [16], and we attribute it to a decrease in the relative abundance of phyla such as Glomeromycota (across all environments), Basidiomycota (in apple orchard and paddy field soils), Mucoromycota (in apple orchard and arid land soils), and Mortierellomycota (in arid land and paddy field soils). Conversely, Peng Bin et al. reported an increase in both the richness and diversity of soil bacterial communities post-cultivation [40,45], which contrasts with our results. This discrepancy may be due to a shift in soil pH toward neutral levels in their study, conditions more favorable for bacterial growth. Furthermore, the formation of ectomycorrhizae with host plants and the reliance of common morel on soil bacteria for nutrient acquisition and hyphal protection could also contribute to increased bacterial species richness [46]. At the phylum level, Proteobacteria and Ascomycota were the dominant bacterial and fungal phyla, respectively, across all environments post-cultivation, consistent with findings by Wu Xiaohui et al. [47]. Specific comparisons to control soils showed increased relative abundance of Bacteroidota (all environments), Proteobacteria (apple orchard and arid land), and Gemmatimonadota (apple orchard and paddy field). Decreases were observed for Actinobacteriota, Planctomycetota, Chloroflexi, and Myxococcota (all environments) and Acidobacteriota and Verrucomicrobiota (arid land and paddy field). For fungi, Chytridiomycota and Ascomycota (all environments) and Ciliophora (apple orchard and paddy field) increased, while Glomeromycota (all environments), Basidiomycota (apple orchard and paddy field), Mucoromycota (apple orchard and arid land), and Mortierellomycota (arid land and paddy field) decreased. Previous research indicates that Proteobacteria possess diverse metabolic types and actively participate in various biogeochemical cycles. Among them, alpha- and beta-class bacteria engage in symbiotic nitrogen fixation within plant root nodules, whereas gamma-class bacteria are involved in the degradation of various organic compounds [48]. The observed increase in Proteobacteria is therefore hypothesized to be associated with the concurrent rises in alkaline-hydrolyzable nitrogen and organic matter across the three environmental conditions examined in this study. Bacteroidota harbor nitrogen-fixing genes (nif), Acidobacteria are adapted to oligotrophic and dry conditions through protein secretion [40,49], and Actinobacteria exhibit antifungal activity and promote plant growth, indirectly benefiting common morel [50]. Thus, the differential shifts in these beneficial bacterial phyla across environments provide a theoretical basis for site-specific common morel cultivation. At the genus level, *Sphingomonas* was the dominant bacterial genus across environments, aligning with Cheng’s research [51]. Relative to controls, increases were noted for *Sphingomonas* and *Gemmatimonas* in all environments and *Flavobacterium*, *Flavisolibacter*, and B*ryobacter* in PG. *Sphingomonas* can fix nitrogen, secrete growth hormones like IAA and gibberellins to promote plant and hyphal growth and suppress pathogens [52,53]. *Bryobacter* contributes to soil carbon cycling [54]. These increases are likely associated with the observed rise in soil nitrogen and organic matter content in our study.

## 5. Conclusions

Bacterial and fungal communities in soil following Common morel cultivation were studied in three environments: apple orchards, arid land, and paddy fields. The results indicate that common morel cultivation enhanced various soil nutrient components. Furthermore, changes in soil nutrients were correlated with shifts in microbial community diversity and dominant populations. Regarding cultivation under different conditions, the patterns of change in soil chemical properties, enzyme activities, and microbial communities showed convergent characteristics between the apple orchard and arid land sites. This convergence suggests that soil parameters following a consistent trend can be managed through systematic regulation. In contrast, changes in most indicators following cultivation in paddy fields were distinct, necessitating differentiated intervention strategies. These findings provide a theoretical basis for selecting cultivation environments and implementing scientific management practices for the common morel.

## Figures and Tables

**Table 1:** Comparison of Soil Chemical Properties After Cultivating Common Morel in Different Environments.

Group	TN (g/kg)	HN (mg/kg)	AK (g/kg)	Organic Matter (g/kg)	TP (g/kg)	TK (g/kg)	AP (mg/kg)	PH	EC (μS/cm)
PG-CK	1.62 ± 0.03 a	127.79 ± 22.35 a	136.17 ± 4.96 b	32.10 ± 1.25 b	0.96 ± 0.03 b	13.17 ± 0.25 b	11.22 ± 1.12 b	6.19 ± 0.06 b	65.43 ± 4.37 b
PG-1-A	1.40 ± 0.07 b	132.32 ± 11.75 a	482.54 ± 7.59 a	35.15 ± 0.85 a	1.41 ± 0.01 a	14.32 ± 0.18 a	38.92 ± 2.44 a	7.07 ± 0.07 a	157.33 ± 6.35 a
HD-CK	1.11 ± 0.09 a	123.26 ± 15.85 b	187.06 ± 3.57 b	37.73 ± 1.26 a	1.55 ± 0.01 b	9.51 ± 0.22 b	5.40 ± 0.29 b	6.19 ± 0.06 b	168.97 ± 2.51 a
HD-1-A	1.10 ± 0.08 a	153.62 ± 8.62 a	209.24 ± 8.78 a	38.85 ± 0.78 a	1.58 ± 0.00 a	10.12 ± 0.26 a	23.86 ± 0.61 a	7.07 ± 0.07 a	136.13 ± 5.50 b
DT-CK	1.91 ± 0.10 a	167.77 ± 6.96 b	151.74 ± 4.76 a	41.55 ± 2.52 b	1.19 ± 0.02 a	9.99 ± 0.21 a	8.66 ± 0.83 a	6.64 ± 0.06 a	219.20 ± 5.25 a
DT-1-A	1.64 ± 0.10 b	193.31 ± 8.27 a	161.96 ± 7.40 a	47.53 ± 1.38 a	1.17 ± 0.02 a	9.85 ± 0.07 a	0.97 ± 0.04 b	6.41 ± 0.09 b	170.70 ± 1.42 b
PG-CK CV (%)	1.85	6.52	3.64	32.0951	2.76	1.88	10.02	0.98	6.67
PG-CK CV (%)	5.00	8.88	1.57	35.1524	0.41	1.26	6.28	1.04	4.04
HD-CKCV (%)	8.34	12.86	1.90	37.7326	0.65	2.35	5.34	1.77	1.49
HD-1-ACV (%)	6.81	5.61	4.19	38.8475	0.00	2.54	2.54	1.81	4.04
DT-CKCV (%)	5.02	4.15	3.13	41.5484	1.28	2.07	9.63	0.91	2.39
DT-1-ACV (%)	6.12	4.28	4.57	47.5249	2.76	0.77	3.67	1.42	0.83
F value	46.97 **	20.64 *	1229.80 ***	40.44 **	779.99 ***	291.71 ***	426.68 ***	36.12 ***	370.72 ***

Note: Excel and SPSS 20 were used for statistical analysis. Different letters show significant difference at *p* < 0.01 by the LSD method of a one-way ANOVA. Abbreviations: PG-CK, soil from apple orchards without common morel cultivation; PG-1-A, soil from apple orchards with common morel cultivation; HD-CK, soil from dryland fields without common morel cultivation; HD-1-A, soil from dryland fields with common morel cultivation; DT-CK, soil from paddy fields without common morel cultivation; DT-1-A, soil from paddy fields with common morel cultivation; TN, total nitrogen; TP, total phosphorus; TK, total potassium; HN alkali-hydrolyzable nitrogen; AP, available phosphorus, AK, available potassium. The same abbreviations apply hereafter; CV: Coefficient of Variation; * *p* < 0.05, ** *p* < 0.01, *** *p* < 0.001.

**Table 2:** Comparison of Soil Enzyme Activities Following Common Morel Cultivation in Different Environments.

Group	S-CAT (μmol/h/g)	S-PPO (nmol/h/g)	S-UE (μg/d/g)	S-AL (μg/h/g)	S-SC (cmg/d/g)	S-ACP (nmol/h/g)
PG-CK	703.73 ± 2.07 b	305.25 ± 4.12 b	207.63 ± 2.24 b	425.46 ± 2.33 b	16.79 ± 0.46 a	2056.99 ± 4.37 a
PG-1-A	952.50 ± 1.68 a	343.62 ± 2.63 a	356.35 ± 2.10 a	451.28 ± 0.81 a	17.52 ± 0.19 a	1487.90 ± 3.04 b
HD-CK	842.80 ± 4.42 a	259.26 ± 3.47 a	162.29 ± 1.46 a	420.50 ± 2.15 b	13.54 ± 0.18 b	1300.69 ± 8.72 a
HD-1-A	812.76 ± 2.16 b	209.53 ± 2.22 b	132.75 ± 0.95 b	475.38 ± 2.17 a	17.39 ± 0.35 a	1164.14 ± 2.30 b
DT-CK	924.21 ± 1.69 a	323.94 ± 3.92 a	77.24 ± 4.75 a	419.83 ± 2.68 a	19.35 ± 0.48 b	2553.51 ± 2.91 b
DT-1-A	852.55 ± 4.90 b	306.64 ± 3.50 b	70.67 ± 7.45 a	299.88 ± 5.90 b	21.68 ± 0.46 a	2922.08 ± 59 a
PG-CK CV (%)	0.24	1.10	0.88	0.55	2.75	0.21
PG-CK CV (%)	0.14	0.63	0.48	0.18	1.09	0.20
HD-CKCV (%)	0.33	1.09	0.74	0.51	1.30	0.67
HD-1-ACV (%)	0.22	0.86	0.58	0.46	2.03	0.20
DT-CKCV (%)	0.15	0.99	5.02	0.64	2.46	0.11
DT-1-ACV (%)	0.47	0.93	8.61	2.00	2.13	0.16
F value	5553.55 ***	1255.53 ***	4453.73 **	1132.25 ***	156.70 ***	66,364.97 ***

Note: Excel and SPSS 20 were used for statistical analysis. Different letters show significant difference at *p* < 0.01, by the LSD method of a one-way ANOVA. S-AL, Soil Amylase; S-PPO, Solid Polyphenol oxidase; S-CAT, Solid Catalase; S-UE, Solid Urease; S-ACP, Acid phosphatase; S-SC, Solid Sucrase. CV: Coefficient of Variation; ** *p* < 0.01, *** *p* < 0.001.

**Table 3:** Analysis of α-diversity in soil bacterial and fungal communities following common morel cultivation under different environmental conditions.

Rgion	Type	Sobs Index	Chao Index	Ace Index	Shannon Index	PD Index	Pielou Index	Goods_Coverage
16s	PG-CK	8004.33 ± 22.28 a	8491.98 ± 51.60 a	9047.88 ± 45.46 a	11.49 ± 0.38 a	1059.68 ± 28.55 a	0.86 ± 0.02 a	0.96
	PG-1-A	7582.67 ± 17.01 b	8225.39 ± 37.81 b	8821.34 ± 52.54 b	10.81 ± 0.042 a	1000.67 ± 6.75 a	0.84 ± 0.01 a	0.97
	HD-CK	7272.00 ± 19.03 a	7676.72 ± 8.59 a	8176.70 ± 16.44 a	10.61 ± 0.07 a	998.46 ± 15.35 a	0.83 ± 0.01 a	0.97
	HD-1-A	7204.33 ± 4.50 b	7495.17 ± 7.55 b	7968.05 ± 31.35 b	10.70 ± 0.15 a	1021.83 ± 18.63 a	0.83 ± 0.01 a	0.97
	DT-CK	7508.33 ± 7.37 a	7892.27 ± 8.23 a	8385.94 ± 18.36 a	10.98 ± 0.17 a	1014.05 ± 15.26 a	0.85 ± 0.01 a	0.97
	DT-1-A	7066.67 ± 40.50 b	7274.54 ± 17.09 b	7716.12 ± 6.35 b	10.25 ± 0.22 b	981.41 ± 28.06 a	0.80 ± 0.06 a	0.97
	PG-CK CV (%)	4.50	3.50	3.33	2.39	5.52	1.88	0.05
	PG-CK CV (%)	1.36	1.43	1.65	0.85	2.40	0.89	0.29
	HD-CKCV (%)	8.07	7.51	6.91	1.65	7.04	1.11	0.07
	HD-1-ACV (%)	1.50	1.22	0.95	0.84	1.88	0.71	0.03
	DT-CKCV (%)	10.35	9.55	9.15	8.43	12.93	7.31	0.43
	DT-1-ACV (%)	8.48	7.84	7.64	1.60	11.90	0.77	0.38
	F value	1.57 ns	2.90 ns	3.52 *	2.61 ns	0.33 ns	1.98 ns	6.65 *
ITS	PG-CK	1564.00 ± 4.00 b	1787.97 ± 1.93 a	1832.13 ± 7.46 b	7.24 ± 0.20 a	395.48 ± 2.78 a	0.68 ± 0.02 a	1.00
	PG-1-A	1656.33 ± 2.08 a	1891.39 ± 5.77	1915.18 ± 3.91 a	6.64 ± 0.16 b	393.90 ± 2.49 a	0.62 ± 0.62 a	1.00
	HD-CK	1460.33 ± 7.51 a	1627.52 ± 3.77 a	1647.45 ± 5.80 a	7.31 ± 0.15 a	373.83 ± 0.91 a	0.70 ± 0.01 a	1.00
	HD-1-A	1190.33 ± 4.93 b	1423.50 ± 5.51 b	1415.32 ± 12.41 b	6.61 ± 0.31 b	334.73 ± 3.54 b	0.65 ± 0.03 a	1.00
	DT-CK	1245.67 ± 41.93 a	1462.45 ± 36.17 a	1467.79 ± 10.39 a	6.07 ± 0.43 a	327.50 ± 6.16 b	0.59 ± 0.04 a	1.00
	DT-1-A	1266.00 ± 7.55 a	1468.12 ± 11.85 a	1469.65 ± 23.31 a	6.44 ± 0.50 a	339.68 ± 2.97 a	0.62 ± 0.04 a	1.00
	PG-CKCV (%)	2.09	1.59	1.10	2.73	4.70	2.65	0.05
	PG-CKCV (%)	3.41	2.95	3.63	10.64	2.44	10.26	0.02
	HD-CKCV (%)	2.54	1.87	1.76	2.01	2.77	1.67	0.05
	HD-1-ACV (%)	6.26	5.87	5.30	4.73	6.04	5.11	0.00
	DT-CKCV (%)	5.24	6.10	5.57	7.13	4.51	2.65	0.06
	DT-1-ACV (%)	7.68	6.50	6.13	7.76	9.14	10.26	0.02
	F value	26.63 ***	23.39 ***	29.94 ***	3.76 ns	8.30 *	3.22 ns	14.18 ***

Note: Excel and SPSS 20 were used for statistical analysis. Different letters showed significant difference at *p* < 0.01 by the LSD method of a one-way ANOVA; CV: Coefficient of Variation; * *p* < 0.05, *** *p* < 0.001, ns (*p* ≥ 0.05).

## Data Availability

The original contributions presented in this study are included in the article/supplementary material. Further inquiries can be directed to the corresponding author.

## Supplementary Materials

The following are available online at https://www.mdpi.com/article/10.3390/microorganisms14051115/s1, Table S1: Climate conditions during the planting period.

## Abbreviations

The following abbreviations are used in this manuscript: PG Apple orchard soils; HD Dryland soils; DT Paddy field soils; EC Electrical conductivity; TN Total nitrogen; TP Total phosphorus; TK Total potassium; HN Alkali-hydrolyzable nitrogen; AP Available phosphorus; AK Available potassium; S-AL Soil activities of amylase; S-PPO Soil polyphenol oxidase; S-CAT Soil catalase S-UE Soils-urease; S-ACP Soils-acid phosphatase; S-SC Soil sucrase.
